# Acute to Chronic Variants in the Imaging Spectrum of Superficial Siderosis: Case Series and Literature Review

**DOI:** 10.7759/cureus.69491

**Published:** 2024-09-15

**Authors:** Abdul Majith Seeni Mohamed, Seetha Rashi, Anbalagan Malaichamy, Yuvaraj Muralidharan, Sakthi Ganesh Subramonian

**Affiliations:** 1 Radiodiagnosis, Saveetha Medical College and Hospital, Saveetha Institute of Medical and Technical Sciences (SIMATS) Saveetha University, Chennai, IND

**Keywords:** hemosiderin deposition, neurodegenerative disease, subarachnoid hemorrhage, superficial siderosis, susceptibility-weighted imaging (swi)

## Abstract

Superficial siderosis is a rare neurodegenerative disease, related to the deposition of hemosiderin in the central nervous system secondary to recurrent bleeding into subarachnoid space and results in chronic or progressively neurological deterioration. It tends to be due to chronic slow haemorrhages in the setting of previous cranio-spinal trauma or neurosurgery that had been done decades ago. It is important to diagnose the disease as soon as possible because if left untreated it can cause progressive ataxia and deafness, which will require surgical intervention. There are three types of superficial siderosis: Type 1 (the classical infratentorial variant), which is characterised by symmetric deposits in the cerebellum, brain stem, or cranio-cervical junction; type 2 (secondary infratentorial subdural hygroma), an acquired non-communicating secondary supratentorial subdural hygroma (SSDH) subtype manifesting as limited asymmetric fluid collections related to a single intracranial bleeding event and supratentotrial superficial siderosis that represents deposition along cerebral convexities usually due previous hemorrhagic episodes. A singular form is "acute superficial siderosis syndrome," progressing much more rapidly than pure superficial siderosis and due to recurrent haemorrhages. The present case series is intended to describe and illustrate acute and chronic manifestations of superficial siderosis, emphasizing its distinct imaging appearances facilitating early recognition, leading to prompt management.

## Introduction

Superficial siderosis (SS) is a rare and often underdiagnosed neurodegenerative disorder characterized by the pathological deposition of hemosiderin,an iron-storage complex that forms from the breakdown of hemoglobin in red blood cells in the subpial layers of the central nervous system (CNS), primarily due to chronic and recurrent hemorrhages into the subarachnoid space. This condition leads to progressive neurological decline, with symptoms typically emerging years, or even decades, after the initial bleeding event, often linked to previous craniospinal trauma or neurosurgical procedures [1,2]. Despite advances in neuroimaging, particularly the use of MRI with T2-weighted and susceptibility-weighted imaging (SWI) sequences, diagnosing SS remains challenging due to its insidious onset and variability in clinical presentation [3,4]. Many cases are diagnosed only after significant neurological deterioration has occurred, underscoring the need for greater clinical awareness and earlier detection.

The existing literature on SS predominantly focuses on the classical infratentorial variant, with fewer reports addressing the atypical or rapidly progressing forms of the disease, such as acute superficial siderosis syndrome. Furthermore, there is a scarcity of comprehensive case series that showcase the full spectrum of SS, including both acute and chronic manifestations. Detailed imaging studies are essential for improving diagnostic accuracy and guiding timely and effective interventions that could mitigate long-term neurological damage [5,6]. This case series aims to fill this gap by presenting three illustrative cases that presented at Saveetha Medical College and Hospital and demonstrating diverse imaging features of superifical siderosis across its various forms. By highlighting these cases, we seek to underscore the critical importance of early recognition and intervention in SS, a condition that remains largely overlooked yet debilitating in clinical practice.

## Case presentation

Case 1

A 70-year-old male presented to the emergency department with chief complaints of a fall, attributed to incoordination and instability. He reported no history of vertigo or tinnitus. One month prior, he was involved in a road traffic accident (RTA) that resulted in a subarachnoid hemorrhage involving the sulcal space of the right frontal lobe, as well as intraparenchymal frontal and parietal contusions, for which he was treated at a government hospital. He has a history of hypertension and is on regular medication. He denied any history of surgery, and he does not use tobacco products, illicit drugs, or alcohol.

**Figure 1:**
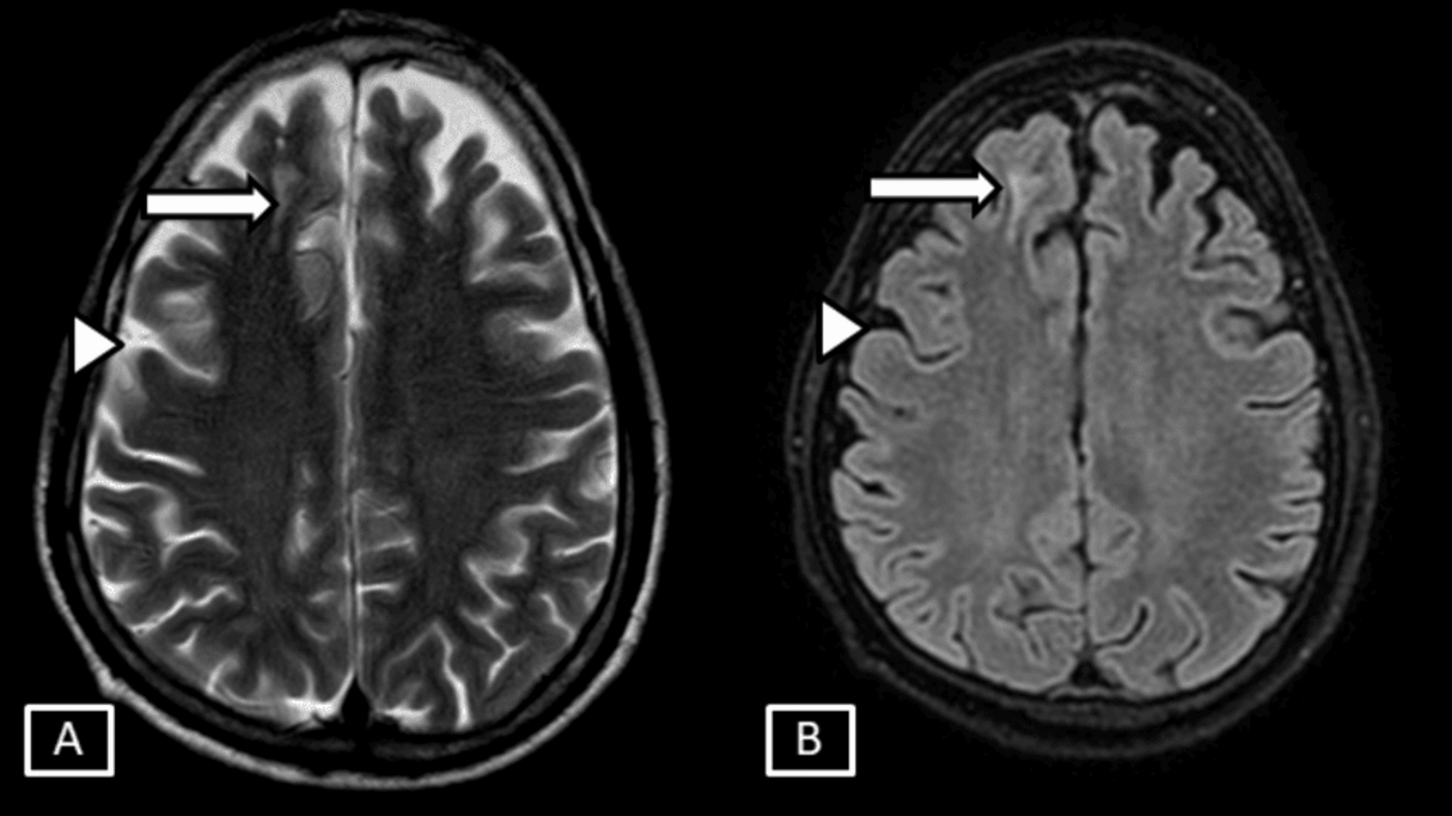
Case 1 MRI T2 axial (A) and T2/FLAIR axial (B). Images show thin linear T2/FLAIR hyperintense signal changes involving the juxtacortical areas of the right frontal lobe represented by the white arrows. Age related brain parenchymal atrophic changes in the form of prominent extra axial sulcal spaces represented by white arrowheads. FLAIR: Fluid Attenuation Inversion Recovery

**Figure 2:**
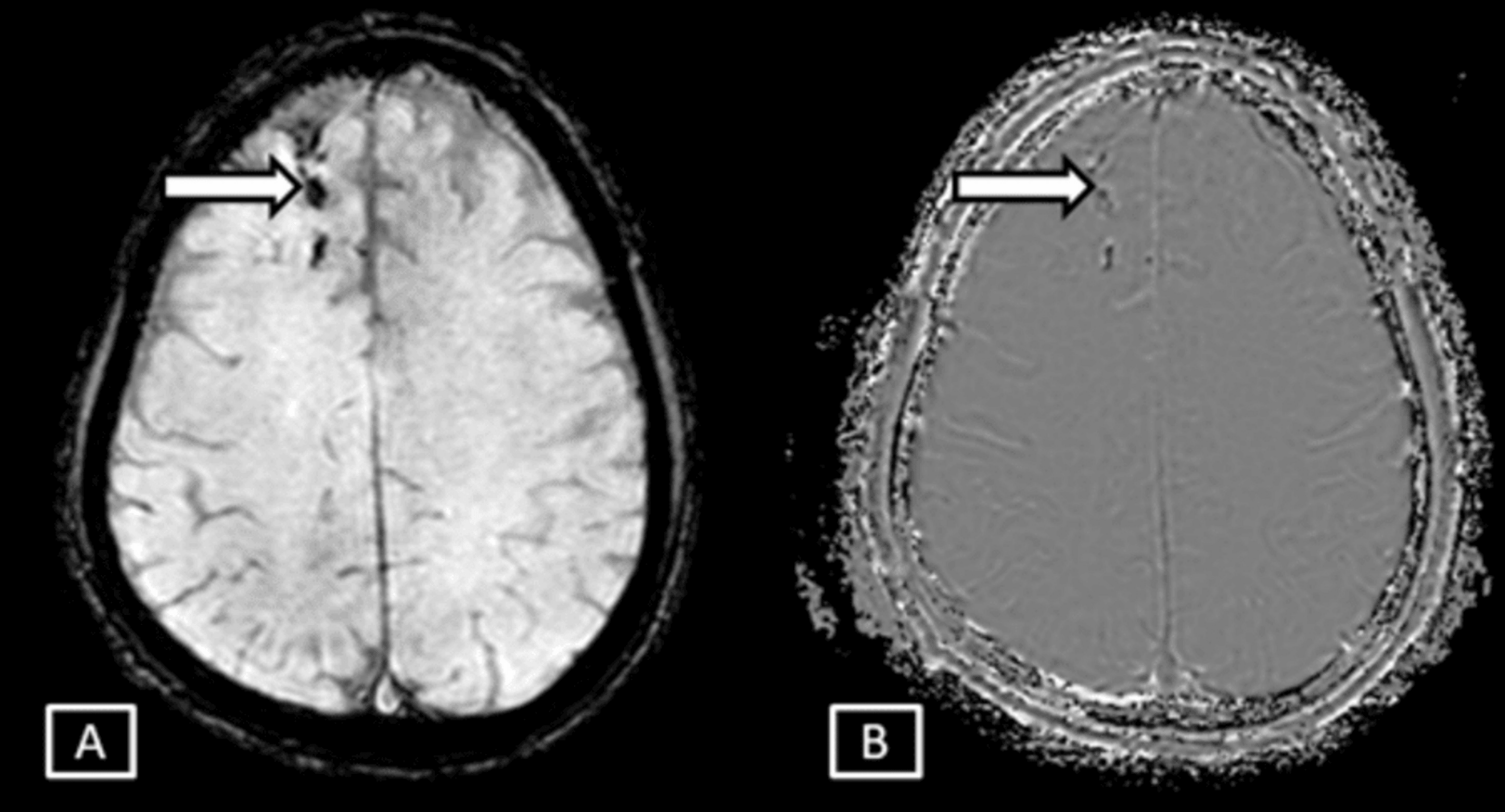
Case 1 MRI axial brain magnitude (A) and phase (B) SWI sequences. Images show corresponding hypointensites in the phase and the magnitude sequences represented by the white arrows. SWI: Susceptibility Weighted Imaging

On examination in the emergency room, the patient was afebrile, normotensive, and maintaining adequate oxygen saturation on room air. He appeared to be in mild distress but was alert and oriented to person, place, and time, with a Glasgow Coma Scale (GCS) score of 15. A neurological examination revealed no focal deficits. His pupils were equal and reactive to light, and the Mini-Mental State Examination (MMSE) indicated normal cognitive function. The otoscopic examination showed a normal tympanic membrane with no abnormalities. While there was no dysmetria on evaluation, the patient exhibited a broad-based, unsteady gait. Orthostatic vital signs were within normal limits. A non-contrast Computed Tomography (CT) scan of the brain revealed no acute abnormalities. The patient was subsequently admitted with a suspicion of stroke, and further evaluation was conducted with Magnetic Resonance Imaging (MRI) of the brain. The MRI showed thin, linear hyperintense signal changes involving the juxtacortical areas of the right frontal lobe on T2-weighted and Fluid Attenuation Inversion Recovery (FLAIR) sequences, with corresponding hypointensities on Susceptibility Weighted Imaging (SWI), suggestive of hemosiderin deposition. The remainder of the brain parenchyma appeared normal, aside from age-related degenerative atrophic changes noted in the form of prominent sulcal and extra-axial CSF spaces (Figures 1, 2).

A diagnosis of superficial cortical siderosis was considered, and given the acute nature of the hemosiderin deposition, that is patient developed symptoms within a month, a diagnosis of Acute Superficial Siderosis Syndrome was made out. The patient was subsequently evaluated in the neurology outpatient department. He was started on iron chelation therapy with deferiprone 500 mg twice daily to reduce the hemosiderin deposition. Citicoline 1000 mg daily was also prescribed to support neural repair and cognitive function. The patient was advised to regularly follow up at intervals to assess the treatment's effectiveness and monitor for any potential side effects.

Case 2

**Figure 3:**
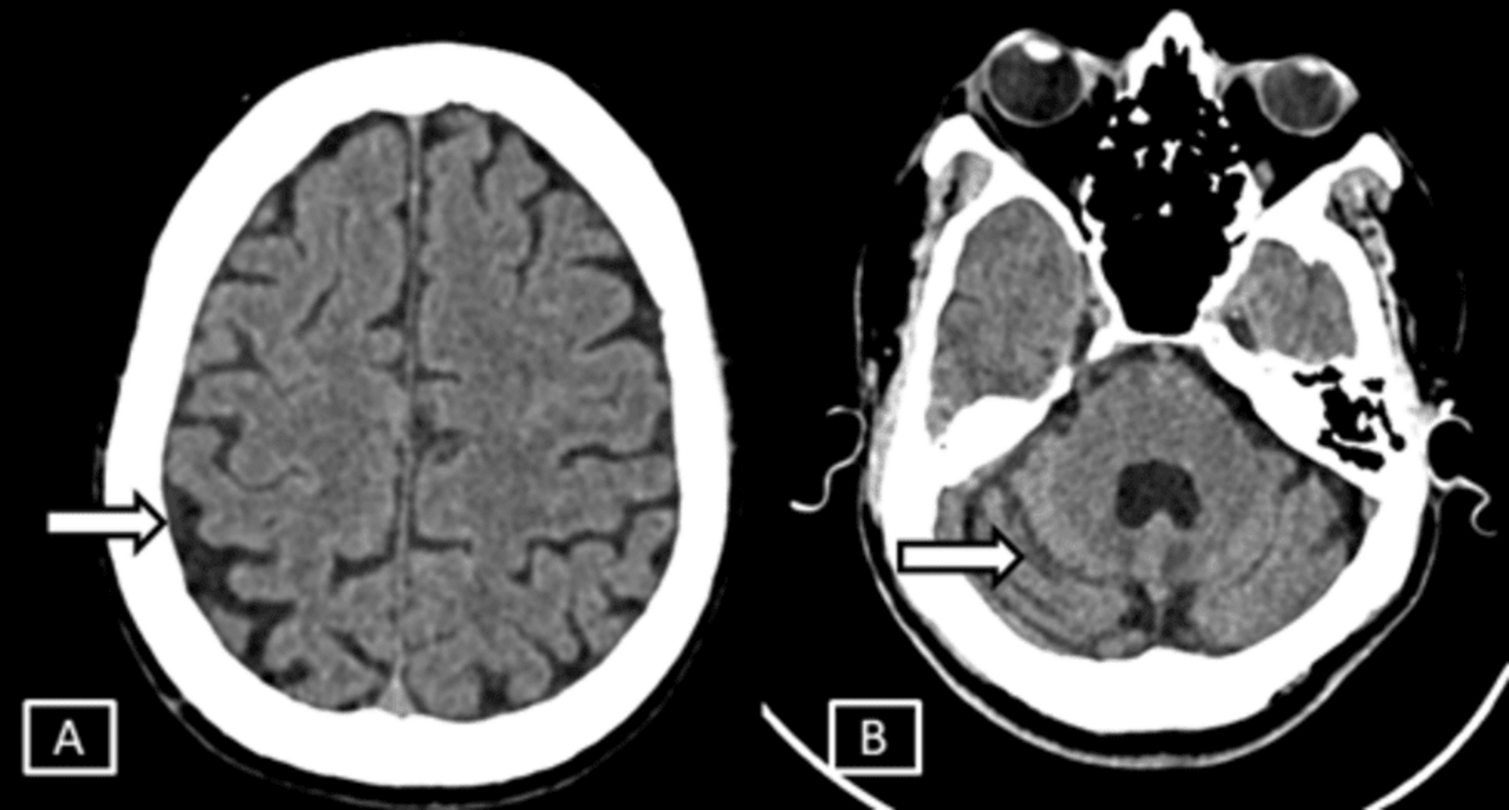
Case 2 CT axial images (A, B) of brain. Images show no significant abnormality except for age-related mild neuroparenchymal atrophic changes in the form of prominent extra-axial sulcal spaces and cerebellar foliae.

A 73-year-old male with no significant past medical history presented to the outpatient department with chief complaints of incoordination and instability while walking. On examination, the patient was afebrile, normotensive, and maintaining adequate oxygen saturation on room air. He was oriented to person, place, and time, with a Glasgow Coma Scale (GCS) score of 15. Neurological examination revealed no focal deficits. His pupils were equal and reactive to light, and the Mini-Mental State Examination (MMSE) indicated normal cognitive function. The otoscopic examination showed a normal tympanic membrane with no abnormalities. Cardiovascular and respiratory examinations were unremarkable, with normal heart sounds and vesicular breath sounds. The abdominal examination also showed no abnormalities. Although there was no dysmetria, the patient exhibited a broad-based, unsteady gait upon evaluation. Orthostatic vital signs were within normal limits. A non-contrast CT scan of the brain revealed no acute abnormalities, aside from mild age-related neuroparenchymal atrophic changes (Figure 3).

**Figure 4:**
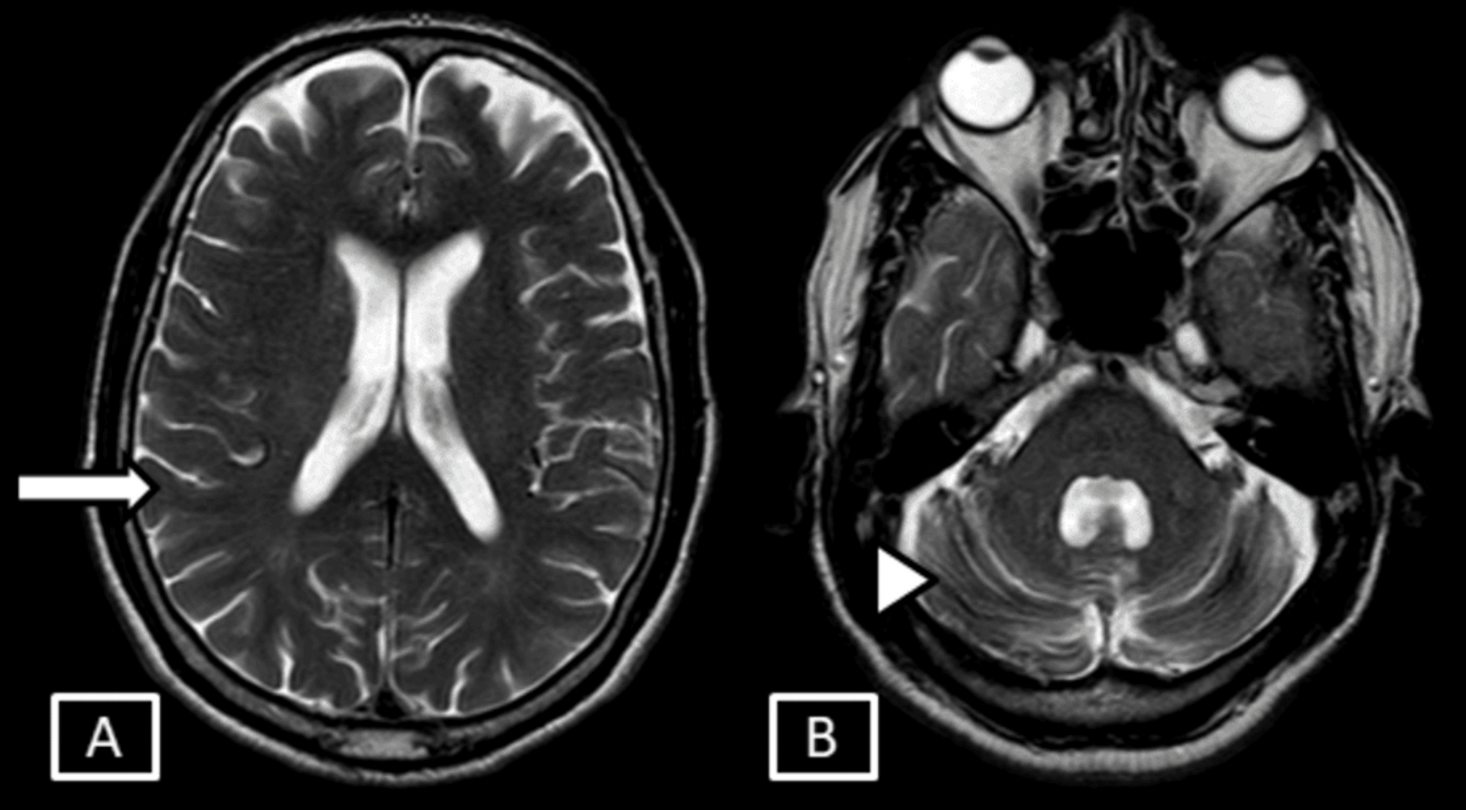
Case 2 MRI brain T2 axial images. Images show mild age-related degenerative changes in the form of prominent sulcal, extra-axial CSF spaces (A) represented by the white arrow and along the cerebellar foliae (B) represented by the white arrowhead. CSF: cerebrospinal fluid

**Figure 5:**
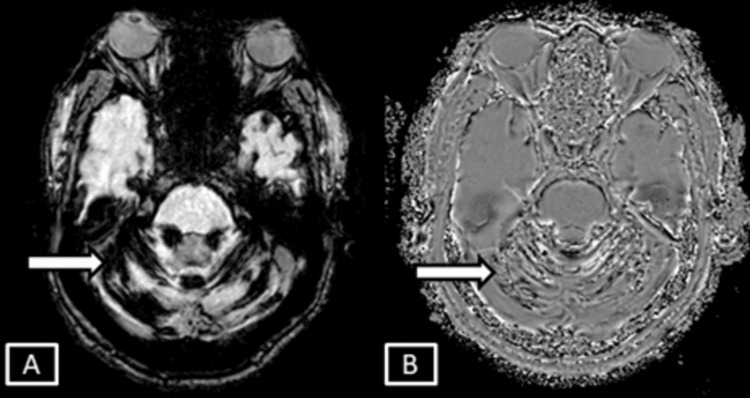
Case 2 MRI brain SWI magnitude (A) and phase (B) sequences. Images show diffuse symmetrical SWI blooming hypointensities seen along the bilateral cerebellar foliae and vermis represented by the white arrows. SWI: Susceptibility Weighted Imaging

**Figure 6:**
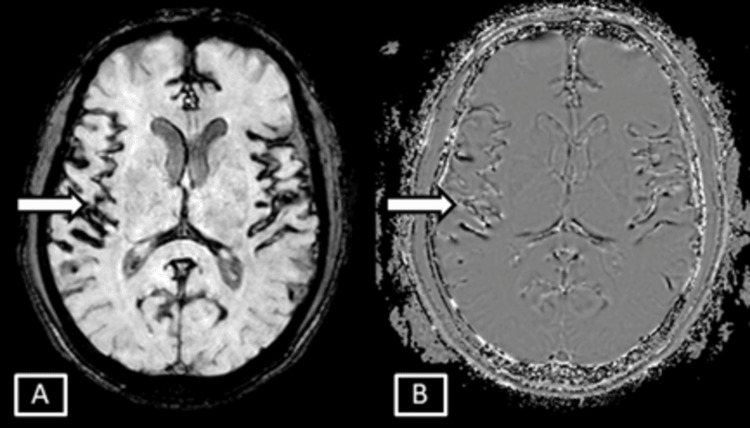
Case 2 MRI brain axial SWI magnitude (A) and phase (B) sequences. Images show symmetrical SWI hypointensites seen involving the sulcal spaces along the cortical surface of bilateral cerebral hemispheres and along the falx represented by the white arrows. SWI: Susceptibility Weighted Imaging

Subsequently, an MRI of the brain was performed, revealing age-related neuroparenchymal atrophic changes in the form of prominent sulcal and extraxial CSF spaces along with diffuse symmetrical linear hypointensities on Susceptibility Weighted Imaging (SWI) sequences. These hypointensities were observed in both the infratentorial and supratentorial compartments, including the bilateral cerebellar folia, vermis, along the falx, and the cortical surfaces of the bilateral fronto-parietal and temporo-occipital lobes, as well as the Sylvian fissures and cisternal spaces (Figures 4-6).

Given the symmetrical deposition of hemosiderin in both the infratentorial and supratentorial compartments, a diagnosis of superficial siderosis of the infratentorial (classical type I) and supratentorial compartments was considered. The patient was referred to the neurology outpatient department for further evaluation and management. The recommended treatment plan included deferiprone for iron chelation and citicoline for neuroprotection. The patient was advised to adhere to this treatment regimen and attend regular follow-up visits for monitoring. However, the patient opted to seek treatment elsewhere, resulting in a loss of follow-up, and we do not have further updates on his condition or treatment outcomes.

Case 3

**Figure 7:**
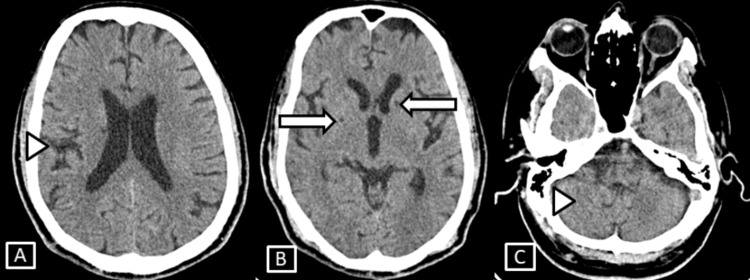
Case 3 CT brain axial images. Images show mild age-related neuroparenchymal atrophic changes involving the sulcal, extra-axial CSF spaces and along the cerebellar foliae (A,C) represented by the white arrowheads. A few chronic lacunar infarcts seen involving the right lentiform nucleus and left external capsule (B) represented by the white arrows. CSF: cerebrospinal fluid

A 60-year-old male presented to the emergency department with complaints of weakness in his left upper limb. He also reported incoordination and instability while walking, with a gradual onset of unsteadiness and difficulty coordinating movements over the past few months, without associated headaches, visual disturbances, or speech difficulties. His medical history includes diabetes and systemic hypertension, for which he is on irregular medications, as well as a 20-year history of alcohol use. He denied any recent falls, trauma, or changes in consciousness, and there was no family history of neurological disorders. On examination, he was afebrile, hypertensive, maintaining adequate oxygen saturation on room air, and oriented to person, place, and time, with a Glasgow Coma Scale (GCS) score of 15. Neurological examination revealed normal cranial nerve function, normal motor function, and intact sensation, but a broad-based, unsteady gait. Cardiovascular and respiratory examinations were unremarkable. A non-contrast CT scan of the brain showed no acute abnormalities, except for mild age-related neuroparenchymal atrophic changes and a few chronic lacunar infarcts (Figure 7).

**Figure 8:**
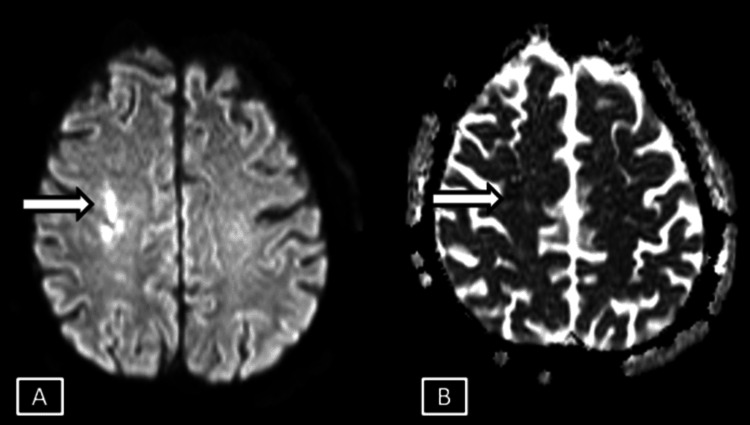
Case 3 MRI brain axial DWI (A) and ADC (B) sequences. Images show a few focal fairly defined areas of diffusion restriction with low ADC signals seen involving the deep white matter areas of right centrum semiovale represented by the white arrows. DWI: Diffusion Weighted Imaging; ADC: Apparent Diffusion Coefficient

**Figure 9:**
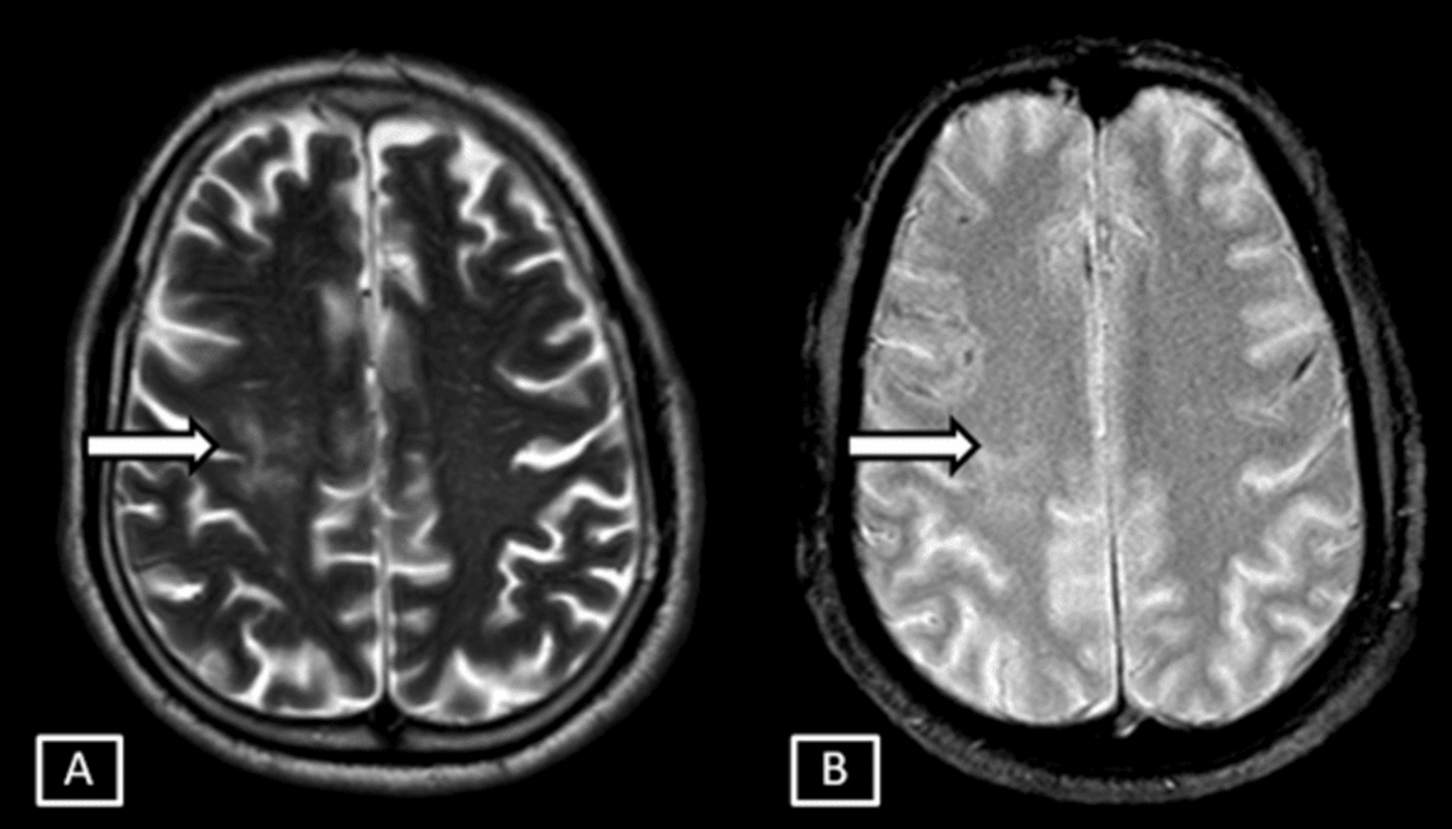
Case 3 MRI brain T2 (A) and GRE (B) sequences. Images show corresponding T2 hyperintense signals with no evidence of any GRE blooming in the area of diffusion restriction suggestive of acute non-hemorrhagic infarct represented by the white arrows. GRE: Gradient Echo

An MRI of the brain subsequently performed revealed age-related neuroparenchymal atrophic changes in the form of prominent extraaxial sulcal and CSF spaces. A few well-defined areas of true diffusion restriction, characterized by hyperintense signals on Diffusion Weighted Imaging (DWI) sequences and hypointense (low) signals on Apparent Diffusion Coefficient (ADC) sequences in the deep white matter of the right centrum semiovale were seen. These areas also appeared isointense on T1-weighted images and hyperintense on T2-weighted images, with no evidence of blooming on Gradient Echo (GRE) sequences, findings likely indicative of an acute non-hemorrhagic infarct (Figures 8, 9).

**Figure 10:**
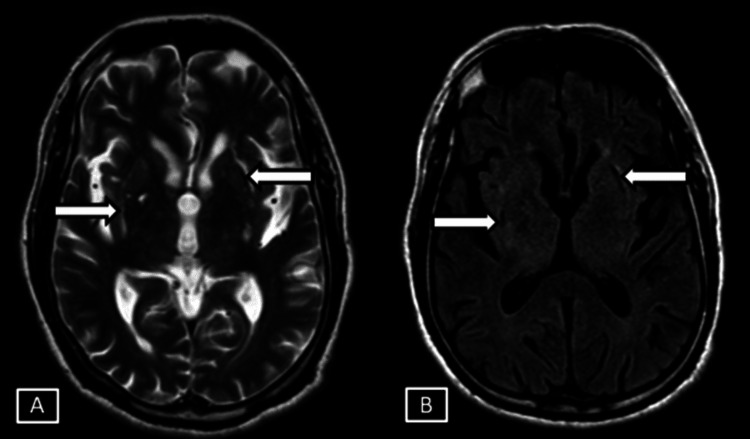
Case 3 MRI brain T2 (A) and FLAIR (B) sequences. Images show chronic infarct changes with surrounding gliosis seen involving the right lentiform nucleus and left external capsule represented by the white arrows. FLAIR: Fluid Attenuation Inversion Recovery

Chronic infarct changes, characterized by CSF signal intensity in all sequences with surrounding gliotic changes, involved the right lentiform nucleus and left external capsule (Figure 10).

**Figure 11:**
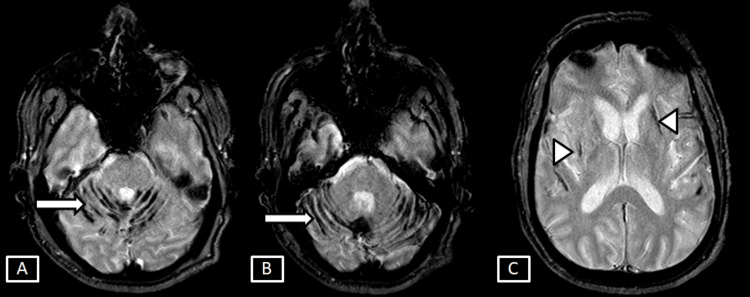
Case 3 MRI brain T2 GRE sequences. Images show symmetrical linear areas of blooming along the bilateral cerebellar foliae and vermis (A, B) represented by the white arrows. Also,  hemosiderin deposition is seen along  the chronic infarct changes of the right lentiform nucleus and left external capsule represented by the white arrowheads. GRE: Gradient Echo

The GRE sequence also revealed symmetrical linear areas of blooming along the bilateral cerebellar folia and vermis, as well as in the regions of chronic infarct changes in the right lentiform nucleus and left external capsule, suggestive of hemosiderin deposition in both infratentorial and supratentorial compartments (Figure 11).

Given the symmetrical deposition of hemosiderin in both the infratentorial and supratentorial compartments, a diagnosis of superficial siderosis of the infratentorial (classical type I) and supratentorial compartments was considered. The patient was subsequently admitted to the ICU by the neurology department for treatment of the acute infarct. During his ICU stay, he was started on antiplatelet therapy with clopidogrel and medications to control his blood pressure. Iron chelators were not initiated immediately due to concerns that they could interfere with the healing process; since superficial siderosis is associated with chronic bleeding, there was a risk that iron chelation could potentially destabilize clots or increase the risk of bleeding. The plan was to consider starting iron chelation therapy two months after the patient had fully recovered. The patient remained in the ICU for four days before being transferred to a regular ward for an additional three days. After this period, he was discharged with instructions to return for a follow-up appointment in two weeks. Unfortunately, the patient did not attend the scheduled follow-up and was lost to follow-up.

## Discussion

Pathophysiology and clinical presentation

Superficial siderosis is an insidious and debilitating neurological disorder caused by chronic subarachnoid hemorrhage, leading to the deposition of hemosiderin in the subpial layers of the central nervous system (CNS). The pathophysiology involves the breakdown of erythrocytes in the cerebrospinal fluid (CSF) and the subsequent release of heme. Heme oxygenase-1 metabolizes heme into free iron, carbon monoxide, and biliverdin, with the free iron being sequestered by ferritin and stored as hemosiderin. This process predominantly affects areas rich in Bergmann glia, such as the cerebellum, which plays a crucial role in synthesizing ferritin in response to prolonged exposure to hemoglobin iron [1, 2].

The clinical manifestations of superficial siderosis encompass a broad range of progressive neurological deficits. The latency period between the initial hemorrhagic event and the onset of symptoms can be extensive, often spanning 10 to 30 years, complicating early diagnosis. Symptoms vary depending on the location of hemosiderin deposits and the extent of neural tissue damage. Approximately 95% of patients experience progressive sensorineural hearing loss, while 88% develop progressive ataxia, characterized by a wide-based gait and balance issues. Additionally, 76% exhibit myelopathy with pyramidal signs. Other symptoms may include mild cognitive impairment, uncontrolled eye movements (nystagmus), poor coordination (dysmetria), difficulty swallowing (dysphagia), spasticity, anosmia, ageusia, anisocoria, bladder or bowel dysfunction, sensory deficits, fine motor dysfunction, neuropathy, headaches, autonomic nervous system dysfunction, dysdiadochokinesia, dysarthria, hyperreflexia, and Babinski signs. The severity of imaging findings does not always correlate with the degree of disability due to the slow progression of the disease [1, 3].

Imaging features

MRI is crucial in the diagnosis and management of superficial siderosis (SS). Key imaging modalities include T2-weighted MRI, Gradient Echo (GRE) sequences, and Susceptibility Weighted Imaging (SWI). SS is characterized by a low signal intensity rim along the surfaces of the brain, brainstem, cerebellum, and spinal cord, due to the paramagnetic properties of hemosiderin. SWI is particularly sensitive in detecting hemosiderin deposits, often revealing more conspicuous findings and identifying additional intracerebral bleeds [4, 5].

High-field MRI further enhances the visibility of hemosiderin deposition, showing marginal hypointensity in various CNS regions, including the cervical cord, medulla oblongata, pons, mesencephalon, and anterior cerebellar surfaces. Key imaging features include widespread peripheral low signal intensity, intraspinal fluid collections, and confluent T2 hypointensity along the subpial surfaces. These patterns are especially visible on GRE or SWI sequences and are critical for accurate diagnosis [6].

Cortical superficial siderosis is a distinct but related form of SS, characterized by hemosiderin deposition limited to the cortical sulci overlying the convexities of the cerebral hemispheres, without involving the cerebellum, brainstem, or spine. It is typically associated with cerebral amyloid angiopathy, an age-related small vessel disease, and can be recognized as linear hypointensities on GRE or SWI MRI. Symptoms range from transient focal neurological attacks and cognitive disturbances to generalized seizures, headaches, and a high risk of intracerebral hemorrhage [7].

Classification of superficial siderosis

Superficial siderosis, while a single pathological entity, can be classified into four distinct types based on clinical and imaging features (Table 1). Classical Infratentorial superficial siderosis (Type 1) is characterized by symmetrical hemosiderin deposition in the infratentorial regions, such as the cerebellum, brainstem, and cranio-cervical junction. Clinically, this type commonly presents with sensorineural hearing loss, progressive ataxia, and myelopathy, with symptoms typically evolving over many years. Imaging reveals symmetrical low signal intensity on T2-weighted MRI, particularly in the cerebellum and brainstem [8]. Secondary infratentorial superficial siderosis (Type 2) occurs following significant intracranial bleeding events, such as subarachnoid or intracerebral hemorrhage, leading to asymmetrical hemosiderin deposition. Symptoms of this type are often localized and correspond to the site of the initial hemorrhage, potentially presenting as focal neurological deficits. MRI findings include asymmetrical hemosiderin deposition, particularly in the cerebellum and other affected infratentorial regions [9]. Supratentorial superficial siderosis (Type 3) involves hemosiderin deposition along the cerebral convexities, often due to prior cortical subarachnoid hemorrhages, and is frequently associated with cerebral amyloid angiopathy (CAA). Clinically, this type may manifest as transient focal neurological episodes, cognitive impairment, and an increased risk of intracerebral hemorrhage, with imaging typically showing hemosiderin deposition in the cerebral convexities, particularly in sulcal regions [7]. Finally, acute superficial siderosis syndrome is characterized by rapid neurological deterioration secondary to recurrent subarachnoid bleeding, making timely diagnosis and management essential to avoid significant neurological decline. Given that each type poses specific diagnostic and management challenges, personalized treatment is required for these patients [10].

**Table 1:** Classification of superficial siderosis

Type	Description	Symptoms	Radiological Features
Classical infratentorial superficial siderosis (Type 1)	Features symmetrical hemosiderin deposition in the cerebellum, brainstem, and cranio-cervical junction.	Hearing loss, ataxia, imbalance, myelopathy	Symmetrical hemosiderin deposition in cerebellum, brainstem, cranio-cervical junction on MRI (low signal intensity on T2-weighted images)
Secondary infratentorial superficial siderosis (Type 2)	Characterized by asymmetrical hemosiderin deposition following significant intracranial bleeding events.	Symptoms arising from subarachnoid or intracerebral haemorrhage	Asymmetrical hemosiderin deposition in affected regions on MRI
Supratentorial superficial siderosis (Type 3)	Involves hemosiderin deposition in the cerebral convexities, often linked to previous cortical subarachnoid haemorrhages and frequently associated with cerebral amyloid angiopathy (CAA).	Transient focal neurological episodes, cognitive impairment, increased risk of intracerebral haemorrhage	Hemosiderin deposition in cerebral convexities on MRI
Acute superficial siderosis syndrome	Progresses rapidly due to repetitive subarachnoid bleeding, requiring prompt diagnosis and intervention.	Severe neurological decline	Rapid and diffuse hemosiderin deposition on MRI

Etiology​ ​​​​​​and affected populations

The causes of superficial siderosis primarily include chronic subarachnoid bleeding and other conditions associated with cerebrospinal fluid (CSF) hypertension, such as chronic sub-occipital hematomas, meningoceles or pseudo-meningoceles that may complicate spinal surgery, venous sinus thrombosis at the base of the medulla oblongata, root avulsions in spinal injuries, and intradural cranial mass lesions like brain tumors, which lead to increased intracranial pressure. Vascular abnormalities and head injuries are also contributing factors. Additionally, capillary regrowth following surgery and nerve damage can result in recurrent CSF bleeding. Hemolysis in the CSF leads to heme overload, which triggers Bergmann glia and microglial cells to produce heme oxygenase-1, releasing toxic free-iron molecules. Astrocytes then secrete ferritin to sequester this free iron, resulting in the formation of insoluble hemosiderin that gravitates towards subpial surfaces, particularly in the infratentorial region and spinal column. Prolonged exposure to free-iron molecules released from hemosiderin is toxic to the underlying neural tissue [3, 11].

Superficial siderosis affects individuals of all races and age groups, with a higher prevalence in males, who are affected three times more often than females. The estimated prevalence of SS is 1 in one million individuals, with approximately 300 diagnosed cases in the U.S. as of 2024. Improved MRI techniques and increased neuro-radiologist awareness have contributed to rising diagnosis rates, although the actual incidence is likely higher [12].

Disorders with similar symptoms and diagnosis 

Superficial siderosis often resembles other conditions such as multiple sclerosis, Parkinson’s disease, or multiple system atrophy, leading to frequent misdiagnosis and delayed treatment [12].The diagnosis of SS can take up to 17 years from the onset of early symptoms, such as tinnitus, dizziness, or phantom odours, due to its slow progression. A thorough review of the patient's long-term medical history, focusing on past surgical procedures, aneurysms, or traumatic events up to 30-40 years prior, is essential. MRI sequencing confirms the diagnosis, with SS characterized by a rim of low signal on the subpial surfaces of the brain, brainstem, and spinal cord, especially on GRE or SWI sequences. Severe forms may also exhibit ependymal hypointensity [6, 13].

Standard therapies

Management of SS starts with identifying and stopping the source of bleeding. A significant percentage of patients have unidentified bleeding sources. CT myelography is preferred for locating dural defects. Treatment options include fibrin glue injections, epidural blood patches for small leaks, and surgical closure of dural defects or active bleed sites. Continued neurological deficits are driven by the release of free iron from existing hemosiderin, making iron chelation therapy essential to remove hemosiderin and potentially halt neural damage and cerebellar atrophy. Deferiprone, an iron chelation agent, shows promise in treating SS by reducing hemosiderin deposition. The use of steroids in superficial siderosis showed only limited and temporary clinical response, suggesting that systematic studies on this treatment are not warranted [9, 13].

Future directions

Although significant advances in imaging, and surgical techniques have be made over the past decades much of our current management strategies for SS still ponder as a result from it long latency period and complex pathophysiology. Further research is required for the validation of sensitive biomarkers to detect an early state of disease, development of new therapeutic agents capable of removing hemosiderin deposition and improvement in surgical techniques with a perspective to prevent recurrent hemorrhage. Interdisciplinarity among neurology, neurosurgery and radiology is essential in pursuing knowledge over superficial siderosis [14]. Table 2 provides a comparative overview of key studies on superficial siderosis, summarizing their focus, principal findings, and implications as extracted from the literature referenced in this review.

**Table 2:** Detailed comparative overview of research on superficial siderosis: study focus, principal findings, and implications CNS: central nervous system; ALS: amyotrophic lateral sclerosis

Study References	Focus of Study	Main Findings	Implications
Willeit J, Aichner F, Felber S, et al. (1992) J. Neurol. Sci. [1]	Report of three cases and literature review on superficial siderosis	Describes clinical features and imaging findings of superficial siderosis in three patients, highlighting the role of MRI in diagnosis.	Early and accurate diagnosis using MRI is critical in managing superficial siderosis and preventing further neurological deterioration.
Vyas S, Giragani S, Singh P, et al. (2011) Ann. Indian Acad. Neurol. [2]	Case report on superficial siderosis	Discusses a case of superficial siderosis diagnosed using MRI and reviews similar cases in the literature.	Reinforces the importance of MRI in diagnosing superficial siderosis, particularly in the Indian population.
Vernooij MW, Ikram MA, Hofman A, et al. (2009) Neurology. [3]	Study on the prevalence of superficial siderosis in the general population	Identified superficial siderosis in the general population using MRI, noting its association with age and other risk factors.	Suggests that superficial siderosis may be more common than previously thought, especially in older adults, and underscores the need for further epidemiological studies.
Gomori JM, Grossman RI, Bilaniuk LT, et al. (1985) J. Comput. Assist. Tomogr. [4]	High-Field MRI imaging of superficial siderosis	Demonstrated the efficacy of high-field MRI in detecting superficial siderosis, providing detailed images of hemosiderin deposition in the CNS.	Highlights the superior diagnostic capability of high-field MRI in identifying superficial siderosis, which can aid in early detection and treatment.
Payer M, Sottas C, Bonvin C (2010) Acta Neurochir. [5]	Case report on superficial siderosis with ALS-like progression	Reports on a patient with superficial siderosis who showed ALS-like symptoms despite successful surgical treatment, indicating secondary progression.	Emphasizes the need for ongoing monitoring in patients with superficial siderosis, even after surgical intervention, to detect potential secondary neurological deterioration.
Grunshaw ND, Blanshard KS, Hussain SSM, et al. (1993) Clin. Radiol. [6]	MRI diagnosis of superficial siderosis	Confirms the role of MRI in diagnosing superficial siderosis, presenting detailed case studies to support its use as a primary diagnostic tool.	Strengthens the evidence for MRI as the gold standard for diagnosing superficial siderosis, particularly in cases with subtle clinical signs.
Charidimou A, Linn J, Vernooij MW, et al. (2015) Brain [7]	Detection and significance of cortical superficial siderosis in cerebral amyloid angiopathy	Explores the relationship between cortical superficial siderosis and cerebral amyloid angiopathy, identifying it as a marker of disease severity.	Suggests that cortical superficial siderosis could serve as a diagnostic marker for cerebral amyloid angiopathy, potentially guiding treatment decisions.
Kharytaniuk N, Cowley P, Sayal P, et al. (2022) Pract. Neurol. [8]	Pathophysiology, clinical features, and management of classical Infratentorial superficial siderosis	Provides a comprehensive overview of the pathophysiology, clinical features, and management strategies for classical Infratentorial superficial siderosis.	Offers valuable insights into the management of classical Infratentorial superficial siderosis, potentially improving patient outcomes through tailored treatment approaches.
Shih P, Yang BP, Batjer HH, Liu JC (2009) Spine J. [9]	Surgical management of superficial siderosis	Discusses surgical strategies for managing superficial siderosis, with a focus on outcomes and potential complications.	Highlights the complexities and challenges of surgically managing superficial siderosis, stressing the need for careful patient selection and postoperative monitoring.
Nanda S, Sharma SG, Longo S (2010) Ann. Clin. Biochem. [10]	Alternative hypothesis on the mechanism of disease in superficial siderosis	Proposes an alternative hypothesis for the pathophysiology of superficial siderosis, suggesting different mechanisms of hemosiderin deposition.	Opens new avenues for research into the underlying mechanisms of superficial siderosis, which could lead to the development of novel therapeutic strategies.
Kumar N (2021) Ann. Neurol. [11]	Clinical review of superficial siderosis	Comprehensive review of the clinical presentation, diagnosis, and management of superficial siderosis, based on recent case studies and literature.	Provides a valuable reference for clinicians managing superficial siderosis, offering updated guidelines on diagnosis and treatment.
Anderson NE, Sheffield S, Hope JKA (1999) Neurology [12]	Clinical presentation of superficial siderosis	Details a case of superficial siderosis with emphasis on clinical presentation and diagnostic challenges, using MRI as the primary diagnostic tool.	Reinforces the importance of recognizing clinical signs of superficial siderosis and confirms MRI as the key diagnostic tool for this condition.
Nunes J, Gomes BC, Veiga R, et al. (2011) Neuroradiol. J. [13]	Case report on superficial siderosis	Case report describing the diagnosis and clinical management of superficial siderosis using MRI, with a focus on the neuroradiological aspects.	Highlights the role of neuroradiology in diagnosing and managing superficial siderosis, suggesting MRI as a vital tool in both diagnosis and treatment planning.
Kumar N (2007) Arch. Neurol. [14]	Review on superficial siderosis	Provides an overview of superficial siderosis, including its clinical features, imaging findings, and potential treatment options, focusing on long-term outcomes.	Suggests that long-term management strategies are essential for patients with superficial siderosis to prevent progressive neurological damage and improve quality of life.

## Conclusions

In conclusion, advancements in radiological techniques, particularly the refinement of MRI modalities such as T2-weighted, Gradient Echo (GRE), and Susceptibility Weighted Imaging (SWI), have significantly enhanced the diagnostic accuracy and management of superficial siderosis (SS), a rare neurodegenerative disorder characterized by hemosiderin deposition in the central nervous system due to chronic subarachnoid hemorrhage. These imaging advancements have allowed for better identification of hallmark low-signal intensity rims, enabling thorough evaluation of CNS involvement, timely interventions, and effective monitoring of disease progression. High-field MRI has become indispensable for assessing disease severity and guiding therapeutic decisions. Moving forward, early detection can be improved through interdisciplinary collaboration between neurologists, radiologists, and neurosurgeons, coupled with AI-driven analysis of imaging databases to detect subtle early-stage signs. Regular MRI screenings for high-risk individuals and fostering international research cooperation can further enhance diagnostic precision. Raising awareness among at-risk individuals to seek early evaluations for subtle symptoms is also crucial. Future research should focus on refining these imaging modalities and developing personalized treatments such as surgical interventions and iron chelation therapy, ultimately improving patient outcomes and addressing the complexities of SS with greater accuracy and effectiveness.
